# Amyloid-beta antibody binding to cerebral amyloid angiopathy fibrils and risk for amyloid-related imaging abnormalities

**DOI:** 10.1038/s41598-024-61691-2

**Published:** 2024-05-13

**Authors:** Linda Söderberg, Malin Johannesson, Eleni Gkanatsiou, Patrik Nygren, Nicolas Fritz, Olof Zachrisson, Adeline Rachalski, Anne-Sophie Svensson, Emily Button, Giacomo Dentoni, Gunilla Osswald, Lars Lannfelt, Christer Möller

**Affiliations:** 1 BioArctic AB, Warfvinges väg 35, 112 51 Stockholm, Sweden; 2 Department of Public Health/Geriatrics, Uppsala University, 751 85 Uppsala, Sweden

**Keywords:** Alzheimer’s disease, Amyloid, ARIA, CAA, Immunotherapy, Alzheimer's disease, Alzheimer's disease

## Abstract

Therapeutic antibodies have been developed to target amyloid-beta (Aβ), and some of these slow the progression of Alzheimer’s disease (AD). However, they can also cause adverse events known as amyloid-related imaging abnormalities with edema (ARIA-E). We investigated therapeutic Aβ antibody binding to cerebral amyloid angiopathy (CAA) fibrils isolated from human leptomeningeal tissue to study whether this related to the ARIA-E frequencies previously reported by clinical trials. The binding of Aβ antibodies to CAA Aβ fibrils was evaluated in vitro using immunoprecipitation, surface plasmon resonance, and direct binding assay. Marked differences in Aβ antibody binding to CAA fibrils were observed. Solanezumab and crenezumab showed negligible CAA fibril binding and these antibodies have no reported ARIA-E cases. Lecanemab showed a low binding to CAA fibrils, consistent with its relatively low ARIA-E frequency of 12.6%, while aducanumab, bapineuzumab, and gantenerumab all showed higher binding to CAA fibrils and substantially higher ARIA-E frequencies (25–35%). An ARIA-E frequency of 24% was reported for donanemab, and its binding to CAA fibrils correlated with the amount of pyroglutamate-modified Aβ present. The findings of this study support the proposal that Aβ antibody-CAA interactions may relate to the ARIA-E frequency observed in patients treated with Aβ-based immunotherapies.

## Introduction

The neuropathological changes associated with Alzheimer’s disease (AD) include features composed of aggregated amyloid-beta (Aβ) peptides. Amyloid plaques are mainly found in the grey matter of the brain and primarily consist of aggregated Aβ42^1^. An individual with AD and plaque pathology often (but not always) has cerebral amyloid angiopathy (CAA), which is caused by Aβ accumulation alongside or within the cerebral vasculature^2^. This is a major cause of symptomatic lobar intracerebral hemorrhages, as well as smaller focal lesions such as microbleeds and cortical superficial siderosis. CAA can also lead to more widespread cortical and subcortical ischemic changes, including microinfarcts, increased perivascular spaces, and white matter hyperintensities^3–5^. CAA can be differentiated into two distinct subtypes. Type 1 is associated with cortical capillaries, leptomeningeal and cortical arteries, arterioles, veins, and venules, while Type 2 affects leptomeningeal and cortical vessels, but not cortical capillaries^6^. CAA Type 1 contains higher levels of Aβ42, and CAA Type 2 contains higher levels of Aβ40^7–10^. Inheritance of the ε4 allele of the apolipoprotein E (*APOE*) gene is a risk factor for the development of both AD and CAA^11,12^. Individuals with ε4 homozygosity tend to have more severe Type 1 CAA pathology^6^, and a greater likelihood of intracranial hemorrhage caused by CAA^13^.

A number of immunotherapies for AD are currently under development, although only aducanumab and lecanemab have been approved for clinical use. A range of monoclonal antibodies have been designed to target different forms of Aβ; these include aducanumab, bapineuzumab, crenezumab, donanemab, gantenerumab, lecanemab, and solanezumab (Table 1). Amyloid imaging has indicated that some of these antibodies can successfully remove plaque deposits from the brain^14–19^, but clinical trials have also identified adverse events known as amyloid-related imaging abnormalities (ARIA) in a number of patients. ARIA can be associated with edema (ARIA-E) or with microhemorrhage (ARIA-H). The mechanisms underlying these potentially serious adverse events have not been fully elucidated, although they are likely to relate to antibody interactions with aggregated amyloid species within the brain. Antibody-initiated removal of Aβ from neuritic plaques could exacerbate CAA and thus lead to ARIA^20–22^. Another hypothesis proposes that the process is initiated by Aβ antibody binding to the vascular Aβ found in CAA, triggering a series of events that involve vascular amyloid remodeling, increased vessel permeability to fluid or blood components, complement activation, and inflammation^23,24^.

**Table 1:** Antibodies targeting Aβ.

Generic name	Primary Aβ target	CAA binding	ARIA-E rate	Clinical efficacy
Aducanumab	Fibrils/plaques	High	35%^14^	Yes/No^14^
Bapineuzumab	All forms	High	26.7%^15^	No^15^
Crenezumab	All forms	No	0.3%^29^	No^54^
Donanemab	Pyroglutamated Aβ, fibrils/plaques	Low/medium	24%^16^	Yes^35^
Gantenerumab	Fibrils/plaques	High	25%^26,27^	No^28^
Lecanemab	Protofibrils	Low	12.6%^17^	Yes^17^
Solanezumab	Monomers	No	0%^31,32^	No^31,32^

Different incidences of ARIA have been observed in clinical trials, with ARIA-E rates of 30% or greater for aducanumab^14,25^, 26.7% for bapineuzumab^15^, 25% for gantenerumab^26–28^, 24% for donanemab^16^, and 12.6% for lecanemab^17^, while solanezumab and crenezumab have no reported cases of ARIA-E^29–32^. Although the molecular mechanisms underlying these differences are unclear, they are likely to relate to the profiles of antibody binding to different forms of Aβ, and to the ability of the antibody to mobilize vascular amyloid and trigger microglial effector function. Aducanumab and gantenerumab bind preferentially to aggregated fibrillar Aβ, with low affinities for Aβ monomers^33,34^. Donanemab recognizes an N-terminally truncated and pyroglutamated form of Aβ (AβpE3) that is found in amyloid plaque cores^35^. Lecanemab preferentially binds to soluble aggregates of Aβ (protofibrils and oligomers), while also binding moderately to plaques^36^. Bapineuzumab binds to soluble and fibrillar Aβ^37^, crenezumab binds to multiple forms of aggregated Aβ and to monomers^38^, and solanezumab shows selectivity for soluble monomeric Aβ^39^.

The overall objective of this study was to increase understanding of the mechanisms underlying the development of ARIA-E. CAA fibrils were isolated from human leptomeningeal tissue and the binding of a range of therapeutic Aβ antibodies to these fibrils was tested to evaluate whether this related to the frequencies of ARIA-E previously observed in clinical trials.

## Results

### Immunohistochemistry identified CAA in AD leptomeninges

**Figure 1:**
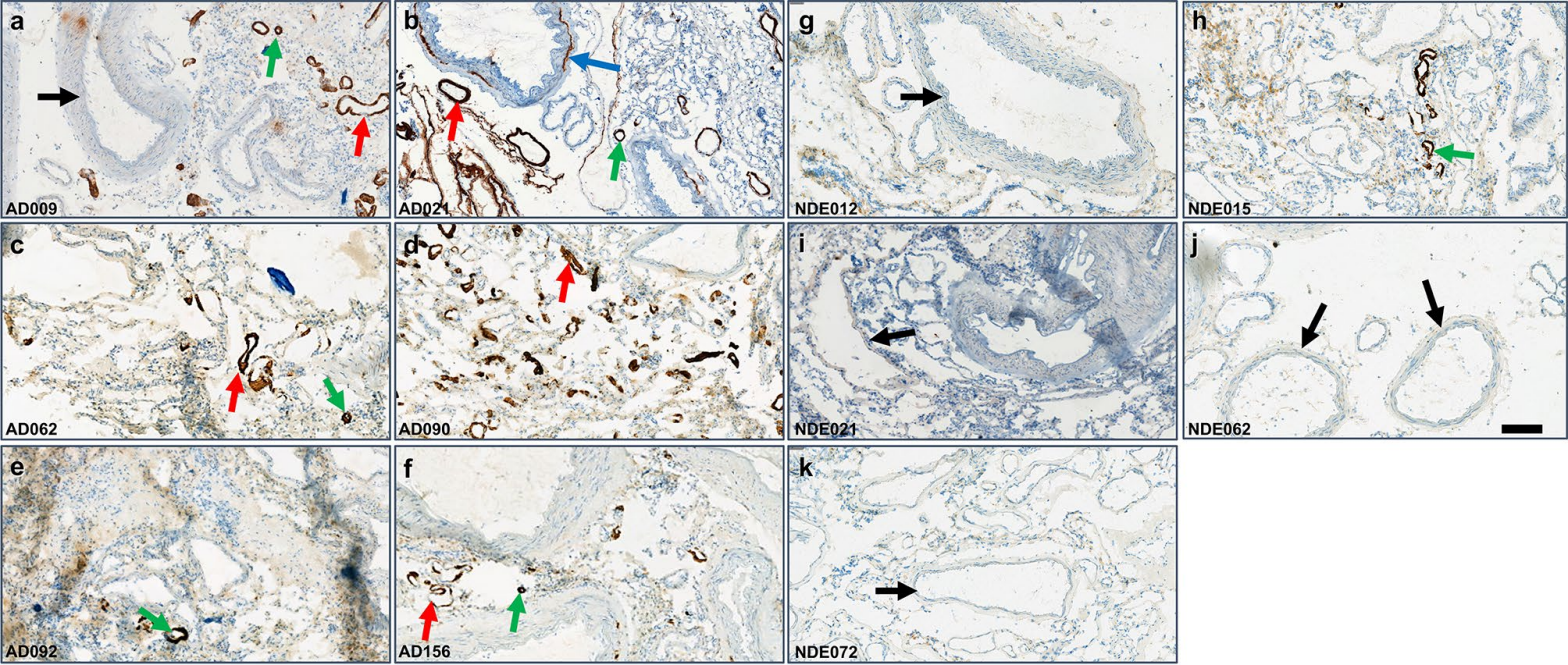
Immunohistochemical staining of total Aβ in fresh frozen leptomeningeal tissue sections. A mixture of 4G8 and 6E10 primary antibodies was used, followed by a horseradish peroxidase 3,3ʹ-diaminobenzidine-based detection system. (**a**–**f**) Representative images of six indicated AD subjects, where green arrows indicate small CAA-positive vessels, red arrows indicate medium-sized CAA-positive vessels, and the blue arrow indicates a large CAA-positive vessel. (**g**–**k**) Representative images of five indicated NDE subjects with no or minimal CAA. Black arrows indicate large CAA-negative vessels. Scale bar = 200 µm.

Total Aβ was stained in fresh frozen leptomeningeal tissue using a mixture of 6E10 and 4G8 antibodies, and representative images are presented in Fig. 1. In all six of the AD subjects investigated (Table 2), immunostaining identified amyloid deposits consistent with CAA in small- to medium-sized vessels. There were individual differences in the extent of immunostaining and some large vessels and arteries were also CAA-positive. Only one of the five non-demented elderly (NDE) subjects investigated showed a low level of CAA-positive small vessels (Fig. 1). Hematoxylin and eosin staining demonstrated that most vessels were intact.

**Table 2:** Study subject demographics.

Case	Diagnosis	Age	Gender	APOE genotype	PMD (h)	Braak stage	Amyloid score	Thal stage	CAA stage, Type
AD009	AD	92	Female	4/3	7:25	4	B	N/A	N/A
AD062	AD	81	Female	4/3	5:30	6	N/A	5	Stage 1, Type 2
AD090	AD	97	Male	4/3	5:10	5	N/A	4	Stage 2, Type 1
AD021	AD	84	Female	3/3	6:33	5	B	N/A	N/A
AD092	AD	72	Female	4/3	6:10	5	N/A	3	N/A
AD156	AD	87	Female	4/3	3:25	4	N/A	3	Stage 2, Type 1
NDE012	NDE	78	Male	3/3	< 17:40	1	0	0	N/A
NDE015	NDE	73	Female	4/4	7:45	1	B	N/A	N/A
NDE072	NDE	76	Female	3/3	7:15	2	0	N/A	N/A
NDE062	NDE	94	Female	3/3	5:50	1	B	N/A	N/A
NDE021	NDE	85	Female	3/2	7:05	1	B	N/A	N/A

*AD* Alzheimer’s disease, *NDE* non-demented elderly, *PMD* postmortem delay, Braak stage indicates a low (0–2), intermediate (3–4) or high (5–6) level of neurofibrillary tangle pathology. Amyloid score (CERAD) indicates no (0) or moderate (B) amyloid plaque load. N/A, information not available. Thal stage^55^ describes the anatomical progression of amyloid plaque pathology where 0 = no amyloid, 4 = basal forebrain and midbrain pathology (beyond stages 1–3 with isocortical, limbic, and basal ganglia involvement), and 5 = pons/medulla oblongata and cerebellar plaque pathology. CAA stage 1 = mild and stage 2 = severe. CAA Type 1 = CAA affecting capillaries and Type 2 = CAA with no capillary involvement.

### Leptomeningeal extracts contain CAA Aβ fibrils

**Figure 2:**
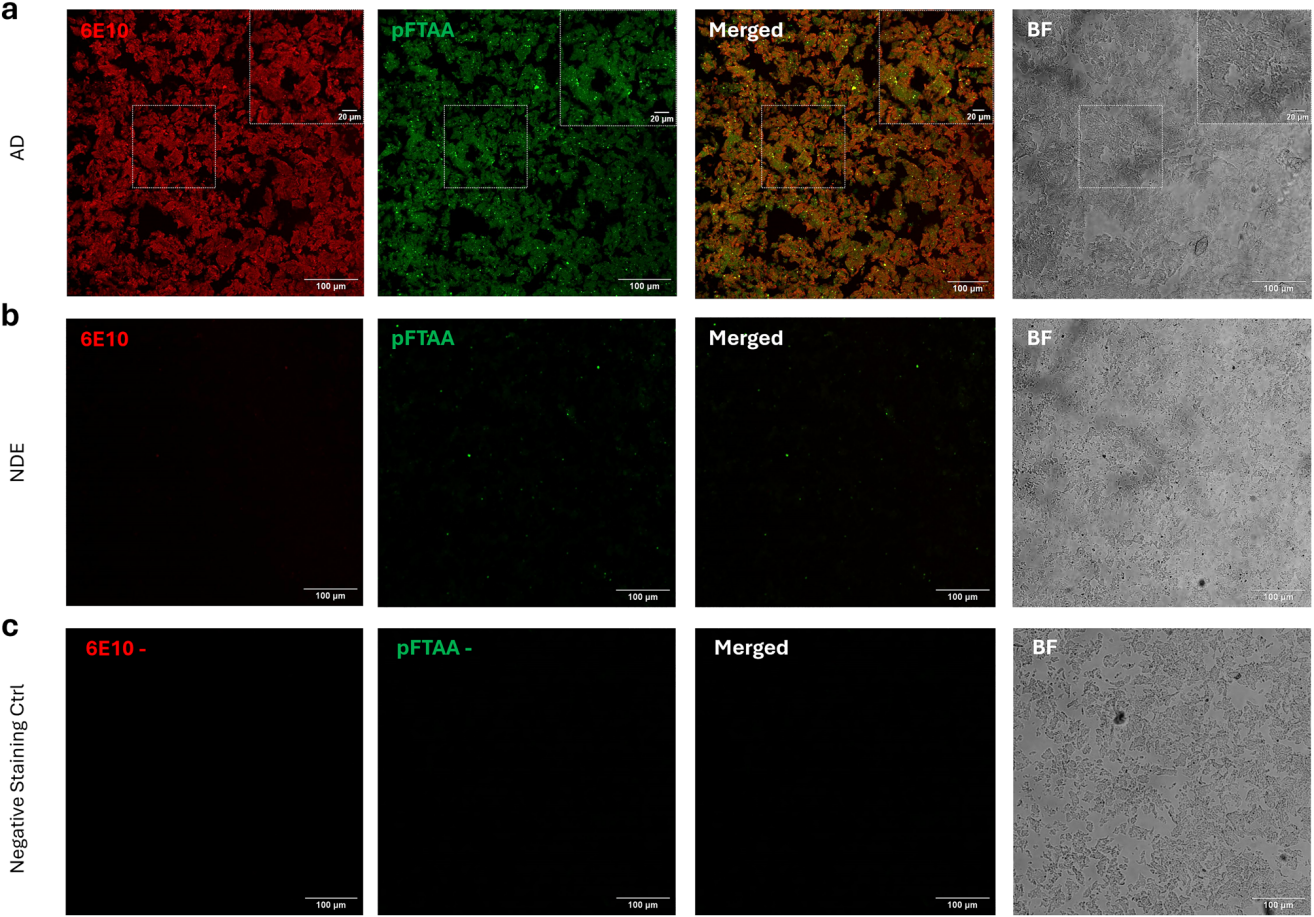
CLSM images of water-extracted leptomeningeal CAA fibrils co-stained with pFTAA (green) and Alexa Fluor^®^ 647-conjugated 6E10 anti-Aβ antibody (red). Merged pFTAA and 6E10 images are shown (yellow represents colocalized staining) and brightfield (BF) pictures were acquired to assess sample morphology. (**a**) Extract from an AD subject (AD009), with boxes at the top right corners showing zoomed-in images. (**b**) Extract from an NDE subject (NDE062). (**c**) AD extract negative staining control (same extract as shown in **a**), with no exposure to pFTAA or 6E10. Scale bars = 100 µm or 20 µm as indicated.

Confocal laser scanning microscopy (CLSM) imaging of leptomeningeal extracts from subjects with AD showed large bright aggregates that were stained by pentameric formyl thiophene acetic acid (pFTAA)^40^ and were Aβ-positive (Fig. 2), consistent with CAA Aβ fibrils.

**Figure 3:**
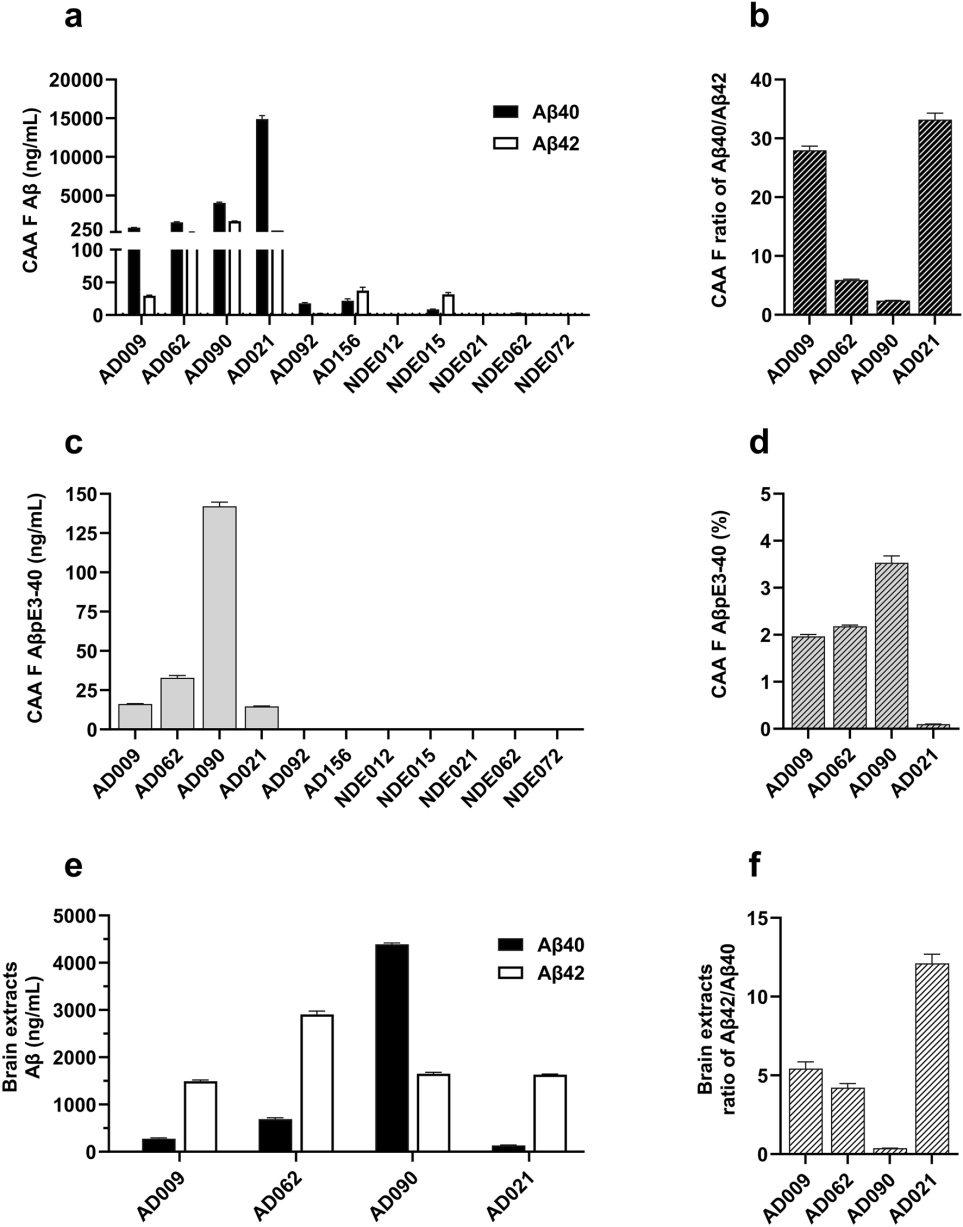
Levels of Aβ peptides in leptomeningeal and temporal cortex extracts. Leptomeningeal CAA fibrils were isolated from subjects with AD (n = 6) and NDE controls (n = 5) and disrupted (70% FA) before V-PLEX^®^ Aβ peptide panel 1 (4G8) assay (MSD) or ELISA (IBL). (**a**) Aβ40 and Aβ42 levels in the indicated subjects; (**b**) Aβ40/Aβ42 ratios for the indicated AD subjects and (**c**) AβpE3-40 levels in the indicated subjects. (**d**) AβpE3-40 level (**c**) expressed as a percentage of total Aβ40 (**a**) for the four AD subjects with measurable levels of both peptides. Data from one representative experiment are shown in (**a**) and (**c**), analyzed in triplicate and presented as mean ± SEM. Temporal cortex insoluble extract Aβ levels were determined by V-PLEX^®^ Aβ peptide panel 1 (4G8) assay. (**e**) Aβ40 and Aβ42 levels and (**f**) Aβ42/Aβ40 ratios in insoluble temporal cortex extracts of the indicated AD subjects.

Immunoassay data revealed that while the leptomeningeal extracts of five NDE subjects had very low or no measurable levels of Aβ40, this peptide was found in the extracts of all six AD subjects (Fig. 3a). Aβ40 was present at high levels in four of these, indicating the presence of CAA Aβ fibrils. In the other two AD subjects (AD092 and AD156), the extraction procedure was repeated on additional samples of leptomeningeal tissue and Aβ levels were reanalyzed. These analyses confirmed that these individuals had low levels of Aβ40 in their leptomeningeal extracts. Although each extract was enriched for Aβ40 relative to Aβ42, there were individual differences in this ratio (Fig. 3b), particularly in AD090 where a moderate amount of Aβ42 was present. Analysis of AβpE3-40 in CAA fibril preparations detected measurable levels in the AD subjects that had robust levels of Aβ40 (Fig. 3c), with some individual variation in the contribution of this peptide to total Aβ40 (Fig. 3d).

Temporal cortex samples from the AD subjects with confirmed high levels of leptomeningeal CAA were also analyzed to determine their insoluble extract levels of Aβ40 and Aβ42 (Fig. 3e). In all four subjects, these cortical insoluble extracts showed measurable levels of both Aβ40 and Aβ42 that were consistent with those measured in other AD samples (in-house data, not shown). With the exception of AD090, Aβ42 levels were higher than Aβ40 levels in these cortical insoluble extracts (Fig. 3f), consistent with plaques being enriched for Aβ42. These data indicated that the leptomeningeal CAA extracts were not contaminated by Aβ derived from parenchymal amyloid plaques.

**Figure 4:**
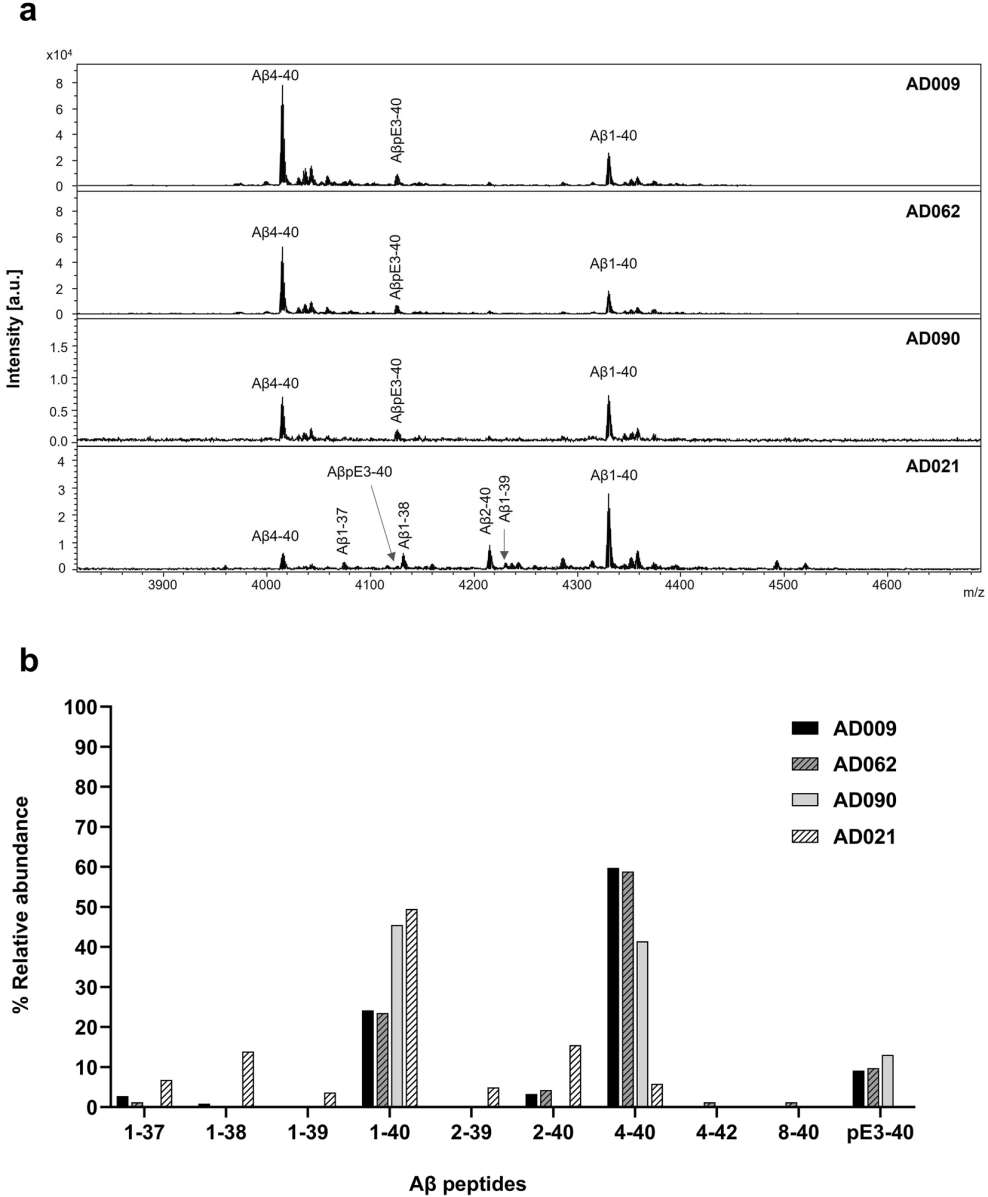
Representative MALDI mass spectra for water-extracted CAA fibrils from leptomeningeal tissues of the indicated AD subjects. Prior to MALDI, CAA fibrils were immunoprecipitated with Dynabeads M-280 sheep anti-mouse beads coupled to 6E10 or 4G8 antibodies. (**a**) Mass spectra for the indicated study subjects, showing Aβ-assigned peaks that were analyzed using an in-house script that matched the masses of the observed peaks to those of known Aβ peptides. (**b**) The relative abundance of Aβ peptides identified in each sample.

In the four AD subjects with high levels of Aβ, immunoprecipitation matrix-assisted laser desorption/ionization time-of-flight mass spectrometry (IP-MALDI) analyses of CAA fibrils identified both full-length and truncated Aβ peptides, including pyroglutamated forms (Fig. 4). Although this approach identified a unique Aβ peptide pattern in each subject, Aβ1-40 and Aβ4-40 were the most abundant peptides in the majority of cases, accounting for > 80% of total Aβ. Other Aβx-40 species, including AβpE3-40, were detected at lower relative levels in the CAA fibrils. The highest relative level of Aβ1-40 was observed in AD021, where it accounted for 50% of the total Aβ signal. This subject had a much lower level of Aβ4-40 than the other AD subjects, and relatively higher levels of C-terminal truncated Aβ species ending at positions 37, 38 or 39. The amounts of Aβx-42 peptides identified in CAA fibrils using this approach were very low, indicating that their levels were below the detection limit. Our analysis of the water-insoluble pellet fractions that remained after water extraction (Supplementary Fig. 1a) identified additional peptide peaks, probably because these samples were more concentrated than the water-extracted fibril fractions. However, the mass spectra showed that the pellet fraction and the CAA fibril preparations from the same individual had very similar peptide patterns (Supplementary Fig. 1b).

### Antibodies show different levels of binding to CAA Aβ fibrils

Three experimental approaches were used to examine the interactions between Aβ antibodies and CAA Aβ fibrils isolated from human leptomeninges.

**Figure 5:**
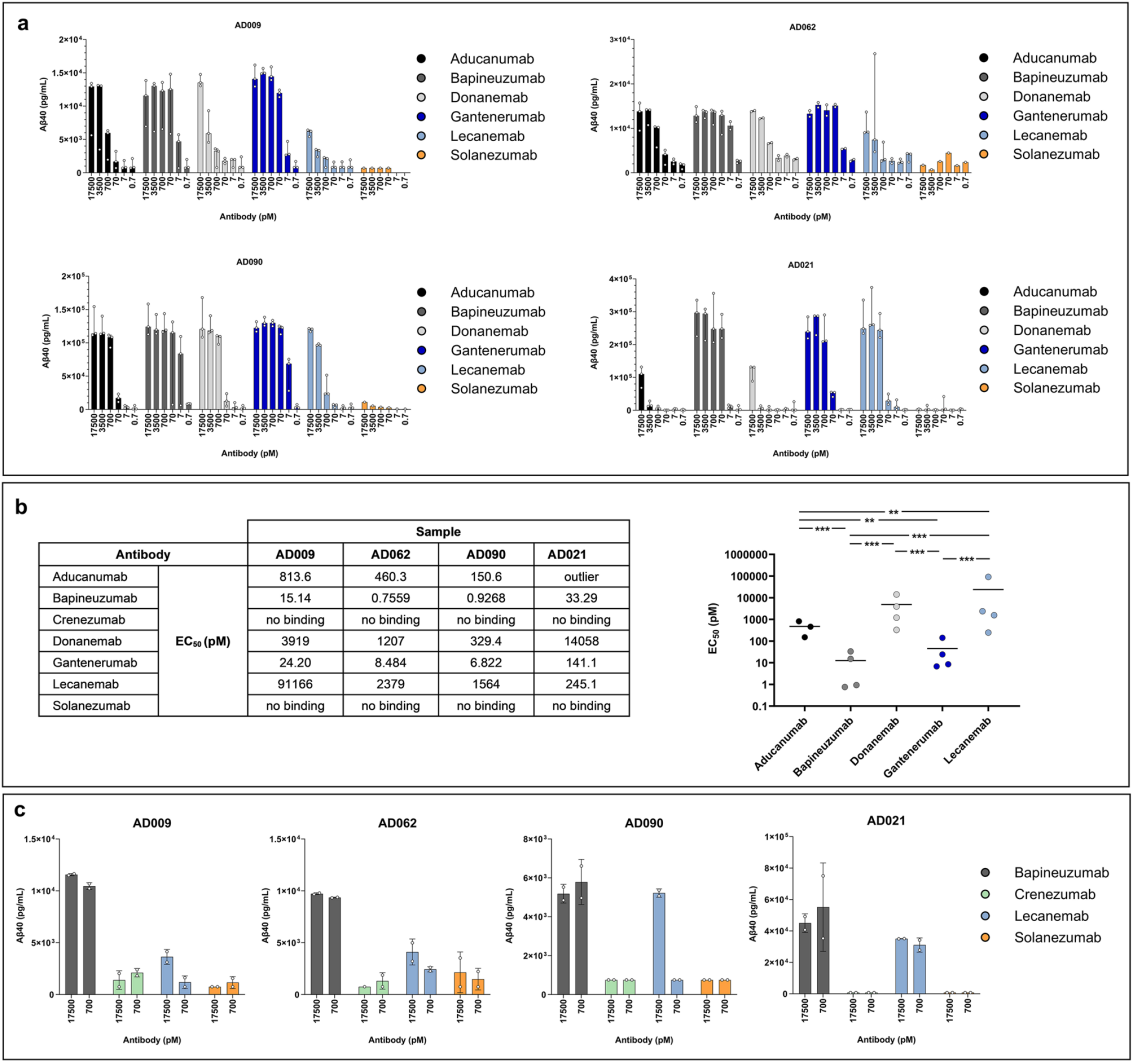
IP analysis of CAA Aβ fibrils extracted from AD leptomeningeal tissues. CAA Aβ fibrils were isolated from the indicated four AD subjects and IP was conducted using the indicated antibody concentrations before eluate analysis using the V-PLEX^®^ Aβ peptide panel 1 (4G8) kit. (**a**) Eluate Aβ40 levels are presented as the median ± range, with each open circle representing an individual experiment. (**b**) The estimated EC_50_ values for each individual and antibody (left-hand panel) and comparison of mean EC_50_ values for the indicated antibodies (right-hand panel). A statistically significant ANOVA (F_4.8_ = 58.93, *p* = 0.00001) was followed by Tukey’s unequal N post-hoc testing; **p < 0.01, ***p < 0.001. (**c**) Eluate Aβ40 levels (mean ± SD), with each open circle representing an individual experiment.

Immunoprecipitation (IP) was used to quantify the binding of aducanumab, bapineuzumab, donanemab, gantenerumab, lecanemab, and solanezumab to CAA Aβ fibrils isolated from four AD subjects (Fig. 5a). All of the antibodies, except for solanezumab, showed some binding to CAA Aβ fibrils. These data were used to calculate EC_50_ values for each individual subject with respect to each antibody, and these values were log-transformed before comparison by analysis of variance (ANOVA). Statistically significant differences in the average log_10_ EC_50_ values for lecanemab as compared to gantenerumab (*p* = 0.0002), aducanumab (*p* = 0.0096), and bapineuzumab (*p* = 0.0002) were identified and further pairwise comparisons are shown in Fig. 5b. Bapineuzumab and gantenerumab were the strongest binders, followed by aducanumab and donanemab. Lecanemab generally showed weaker binding to the CAA Aβ fibrils, with the exception of fibrils isolated from AD021. A separate experiment was carried out to compare the binding of an additional antibody (crenezumab) with that of lecanemab, bapineuzumab, and solanezumab to fibrils isolated from AD009, AD062, AD090, and AD021. This confirmed that bapineuzumab showed the strongest binding, whereas crenezumab and solanezumab showed no binding to CAA Aβ fibrils (Fig. 5c).

**Figure 6:**
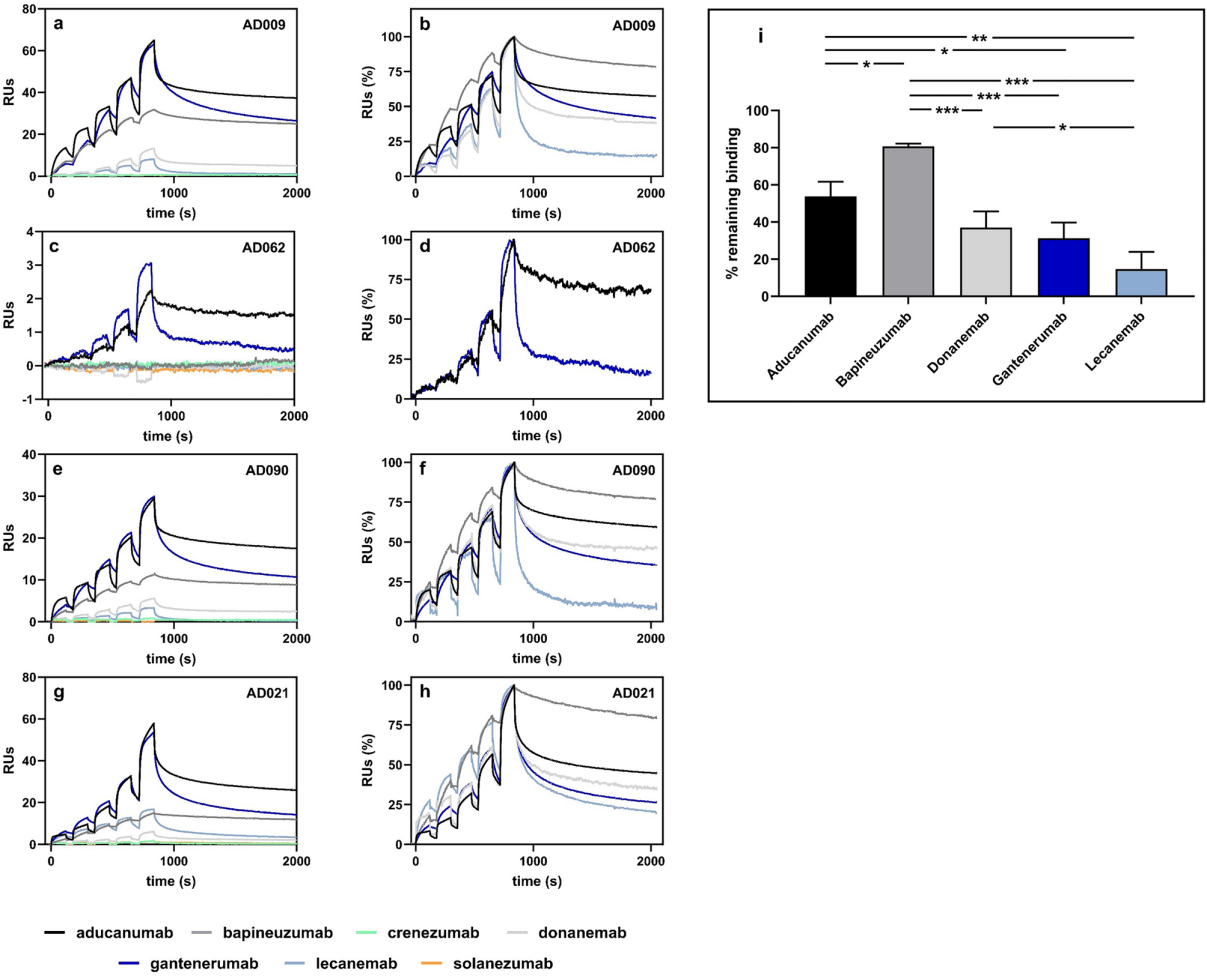
Representative SPR analyses of antibody interactions with CAA Aβ fibrils isolated from the indicated AD subjects. (**a**,**c**,**e**,**g**) Sensorgrams showing the individual responses to increasing concentrations (6.2, 18.5, 55.6, 167, and 500 nM) of the indicated antibodies. On the y-axes, 1 response unit (RU) indicates a weight change of 1 pg/mm^2^ on the sensor chip surface. (**b**,**d**,**f**,**h**) Sensorgrams shown in panels (**a**,**c**,**e**,**g)** were normalized to the highest response observed for each antibody to highlight the difference in dissociation of the antibodies from the antibody-fibril complex during the 20-min dissociation phase. Antibodies that did not produce a binding response (**a**,**c**,**e**,**g)** were omitted from (**b**,**d**,**f**,**h)**. (**i**) Average % of the indicated antibodies that remained bound after the 20-min dissociation phase. One-way repeated ANOVA F_4,8_ = 32.939, *p* = 0.00005. Tukey’s unequal N post-hoc test, *p < 0.05, **p < 0.01, ***p < 0.001. Solanezumab and crenezumab were not included in this analysis.

Surface plasmon resonance (SPR) analyses using CAA Aβ fibrils isolated from four AD subjects identified clear differences between the antibodies (Fig. 6a–h). Crenezumab and solanezumab did not produce any responses when injected over immobilized CAA Aβ fibrils, in agreement with the low fibril binding described by Söderberg et al.^36^. Aducanumab and gantenerumab consistently generated the highest responses, irrespective of which CAA fibril sample was analyzed. These were the only two antibodies that produced responses to CAA Aβ fibrils isolated from AD062, which probably reflected the amounts of fibrils that could be immobilized on the chip, as well as the ability of the antibodies to bind to CAA Aβ fibrils. Lecanemab usually produced much lower responses than aducanumab, bapineuzumab, donanemab or gantenerumab, with the exception of fibrils isolated from AD021, where lecanemab produced a moderate response. To compare these antibody interactions with the CAA Aβ fibrils, we calculated initial kinetic information from the SPR data using the bivalent analyte model. Supplementary Table 1 shows the association rate constant, dissociation rate constant, and dissociation constant (*K*_*D1*_) values for the antibody interactions with CAA Aβ fibrils isolated from four AD subjects. To explore whether there were qualitative differences in the antibodies’ second dissociation rates (*k*_*d2*_), we compared the sensorgrams generated by each antibody-fibril interaction during a 20-min dissociation period (Table 3). This analysis, which only included antibodies that produced SPR responses to CAA fibrils, showed that lecanemab had the lowest residual responses after 20 min. The percentage of lecanemab that remained bound varied between individuals, with the highest level (25%) in AD021. This contrasted with the profiles of aducanumab, donanemab, and gantenerumab, which generally showed less residual binding to fibrils isolated from AD021, as compared with the other cases tested. Bapineuzumab had the most persistent binding after 20 min, with around 80% remaining bound to the CAA Aβ fibrils of each study subject (except AD062). One-way repeated ANOVA of the percentage remaining bound in each subject identified a statistically significant effect (*p* = 0.00005) and Tukey’s unequal N post-hoc test revealed statistically significant differences for lecanemab versus donanemab (*p* = 0.0394), aducanumab (*p* = 0.0011), and bapineuzumab (*p* = 0.0002). Figure 6i provides further pairwise comparisons.

**Table 3:** SPR analysis of antibody binding to CAA Aβ fibrils.

Generic name	AD009		AD062		AD090		AD021	
	Bound (RU)	Remaining (%)	Bound (RU)	Remaining (%)	Bound (RU)	Remaining (%)	Bound (RU)	Remaining (%)
Aducanumab	24.1	62	4.4	46	36.2	59	56.8	48
Bapineuzumab	8.4	82	No binding		18.4	79	15.6	81
Crenezumab	No binding		No binding		No binding		No binding	
Donanemab	3.7	43	No binding		7.3	41	6.2	27
Gantenerumab	23.9	34	12.9	21	35.2	41	51.5	29
Lecanemab	2.4	12	No binding		4.8	7	17.7	25
Solanezumab	No binding		No binding		No binding		No binding	

Bound; average amount of antibody bound to CAA fibrils, expressed as response units (RU).

Remaining; average % remaining bound to CAA fibrils after a 20-min dissociation period.

**Figure 7:**
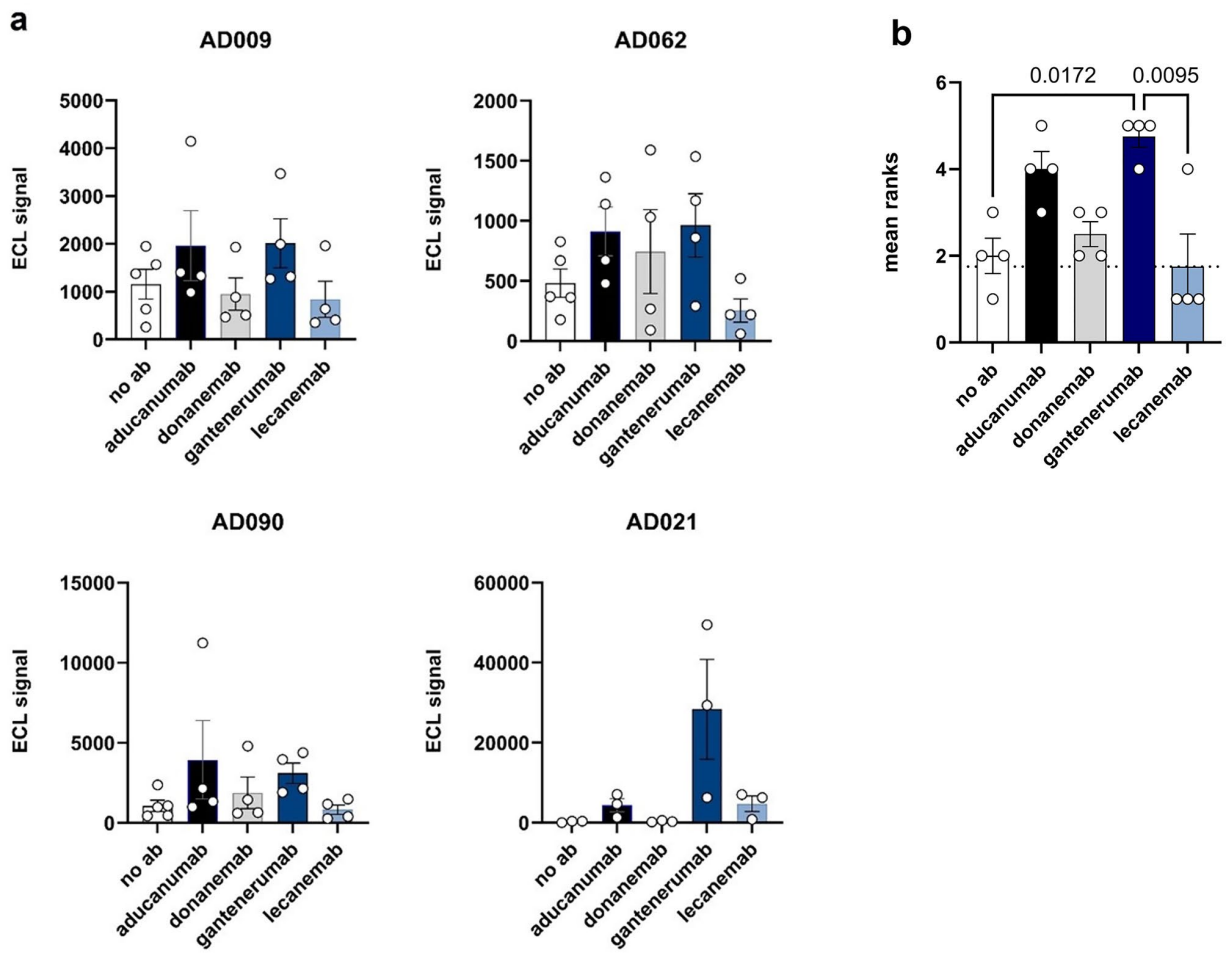
Direct immunoassay of antibody binding to CAA fibrils extracted from AD leptomeninges. (**a**) The signals observed when MSD^®^ plates were coated with CAA Aβ fibrils prepared from the four indicated AD subjects in the absence of antibody (no ab) or in the presence of the indicated antibody (10,000 ng/mL). Bars represent the mean signal ± SEM of four independent experiments, each indicated by an open circle. (**b**) The mean ranking ± SEM of the signals observed when each of the indicated antibodies was individually incubated with CAA fibrils prepared from the four individuals shown in panel (**a**), with individual ranks indicated by open circles. Repeated measures ANOVA, F = 6.60, *p* = 0.0048. Tukey’s post-hoc test *p*-values are shown for each statistically significant pairwise comparison.

The binding of aducanumab, donanemab, gantenerumab, and lecanemab to CAA Aβ fibrils was further tested by direct MSD immunoassay (Fig. 7a). For fibrils prepared from AD009, AD062, and AD090, aducanumab and gantenerumab showed the highest binding, donanemab binding was more variable, and lecanemab binding was similar to that of the negative control (no antibody). A different profile was observed for the CAA Aβ fibrils extracted from AD021, where gantenerumab generated a very high MSD signal and lecanemab and aducanumab produced much lower signals. Notably, donanemab showed no appreciable binding to the fibrils from AD021. To analyze these data further, we ranked the MSD signals measured for each CAA Aβ fibril sample incubated with no antibody, and with each of the four test antibodies, and assigned these a number from 1 (lowest signal) to 5 (highest signal). The mean ranking of each antibody is presented in Fig. 7b, where a higher ranking indicates greater binding to CAA fibrils. This analysis found that gantenerumab had significantly higher rankings than either lecanemab (*p* = 0.0095) or no antibody (*p* = 0.0172). No statistically significant differences were seen between any of the other pairwise comparisons.

## Discussion

This study investigated whether interactions between Aβ antibodies and CAA could potentially influence risk for ARIA-E. We focused on human leptomeningeal tissue as a source of Aβ fibrils derived from CAA. A previous study that isolated and characterized Aβ fibrils from human AD leptomeninges identified structural differences between these naturally occurring fibrils and assemblies of synthetic Aβ peptides^41^. This confirmed the importance of using fibrils that were as similar as possible to those found in the AD brain for the present study. Our characterization of the leptomeningeal extracts from six AD subjects revealed fibrils with beta sheet structure that were primarily composed of Aβ40 peptides. Within-patient comparisons of CAA fibril and insoluble cortical (plaque) extracts found that the plaque extracts were enriched for Aβ42, although AD090 was an exception to this, with fairly similar levels of Aβ40 and Aβ42 in CAA fibril and insoluble cortical extracts. This was consistent with earlier studies of CAA^9,10,42^, as was our finding that the CAA fibrils contained low and variable levels of pyroglutamated Aβ peptides^43^. Our immunohistochemistry (IHC) analyses of CAA were generally consistent with the biochemical data for the same subject, except for AD156, where IHC indicated medium to high levels of CAA while biochemical analyses found very low levels of Aβ. This may reflect regional variations in CAA deposition within the leptomeninges, since two separate tissue samples were used for IHC and biochemical analyses. We did not have CAA stage or Type information for three of the six AD subjects included in the study, which limited our ability to relate our data to neuropathological findings. The leptomeningeal extracts from the other three AD subjects included in the present study contained Type 1 and Type 2 CAA (Table 2). CAA fibrils extracted from AD090 were classified as Type 1 and this may explain the rather low Aβ40/Aβ42 ratio observed in this subject, because Aβ42 is enriched in this CAA sub-type. AD090 also had the highest level of pyroglutamated Aβ, perhaps reflecting the severity of CAA observed. In CAA and in parenchymal plaques, the levels of post-translational modifications such as pyroglutamation and phosphorylation have been shown to increase with amyloid maturation^43^. The five NDE subjects had no CAA reported by the NBB and four of them showed no detectable biochemical or IHC signs of CAA. The one exception was NDE015, with low levels of leptomeningeal extract Aβ and some detectable CAA using IHC. Interestingly, this individual also had plaque pathology (score B) and was homozygous for *APOE* ε4. These findings reinforce the importance of studying several individuals because the differences that we identified in CAA fibril levels and composition, both within and between the study groups, could influence conclusions relating to antibody-CAA interactions.

Two of the six AD subjects analyzed showed low levels of leptomeningeal extract Aβ, indicating that a lower level of CAA was present. In the remaining four AD subjects, three methods were used to assess Aβ antibody binding to the CAA Aβ fibrils. The IP assay allowed us to study this interaction in solution, which is likely to be the optimal approach, and we also studied the concentration dependency of the antibody–CAA fibril interactions. In addition, supporting data were generated using SPR and MSD analyses, which investigated how the antibodies interacted with immobilized CAA fibrils. All three of these methods found that solanezumab and crenezumab, which have no reported cases of ARIA-E^29–32^, showed no or minimal CAA Aβ fibril binding. Lecanemab, which has a lower ARIA-E frequency than aducanumab, bapineuzumab, and gantenerumab, showed much less binding to CAA Aβ fibrils than these antibodies^17,44^. Although a relatively high ARIA-E frequency of 24% has been reported for donanemab^16,35^, we found that it showed variable binding to CAA Aβ fibrils from different individuals, with a preference for fibrils containing higher levels of AβpE3-40. This was exemplified by the increased binding of donanemab to CAA fibrils from AD090, which had a higher ratio of AβpE3-40 to total Aβ40 (3.5%) than those isolated from the other AD subjects. This observation was consistent with in vitro work that we have conducted using synthetic fibrils composed of different ratios of Aβ1-42 and AβpE3-42, which showed that donanemab binding was strongly dependent on the AβpE3-42 content^45^. To address the possibility that inter-individual differences could be caused by experimental variability in the CAA isolation process, we repeated the extraction of CAA fibrils from the same individuals. This revealed consistent Aβ40/42 ratios and percentage AβpE3-40 in fibrils extracted from the same individual, indicating that the leptomeningeal sample selected was unlikely to significantly affect fibril composition or binding properties.

The mechanisms leading to the development of ARIA require further characterization. It is likely that antibody interactions with aggregated amyloid species within the brain are involved and there are two major ways in which these interactions could contribute to ARIA. Antibody binding to cortical neuritic plaques and the associated removal of large quantities of Aβ via perivascular clearance pathways could exacerbate CAA and thus lead to ARIA^2,20–22^. Alternatively, or in addition to this exacerbation of CAA, antibody binding to CAA may be involved. In this context, it is important to note that ARIA-E can also occur in patients who have not been exposed to therapeutic Aβ antibodies. These individuals have CAA-related inflammation (CAA-ri), a rare autoimmune encephalopathy that is associated with increased CSF levels of Aβ autoantibodies^46,47^. The Inflammatory CAA and AD Biomarkers International Network^48^ has enrolled hundreds of patients with CAA-ri and data gathered in this cohort could increase the understanding of the safety of therapeutic Aβ antibodies, which can be regarded as causing iatrogenic CAA-ri^2^. The data generated by the present study and the presence of ARIA-E in individuals with Aβ autoantibodies implies that Aβ antibody-CAA binding may be important in the development of ARIA-E. How this binding event can result in ARIA-E is not known, but it was recently proposed that Aβ antibody-CAA binding can activate the complement system and thus trigger an inflammatory response, resulting in edema due to leakage from the blood vessels^24^. In support of this hypothesis, an IgG1 mutation (K322A) that reduces antibody binding to complement component 1q (C1q) was shown to prevent ARIA-E in an AD mouse model with CAA^49^. This raises the possibility that ARIA-E could be reduced by engineering therapeutic antibodies that do not activate the complement system. The sub-class of antibody employed could also modify risk for this adverse event. For example, no ARIA-E has been reported for crenezumab^29^, which has an IgG4 backbone that results in reduced effector function and lower Fcγ-receptor mediated microglial activation. However, this antibody also failed to achieve clinical efficacy. ARIA-E is more common in individuals carrying an *APOE* ε4 allele; this applies both to patients with CAA-ri and those treated with Aβ immunotherapy. The inheritance of this allele also increases the risk for AD and is associated with a higher CAA burden^11,12^. In the present study, AD021 was the only AD subject who did not have an *APOE* ε4 allele and their CAA fibrils showed some differences in composition and binding properties, as compared with the other AD subjects. A larger number of subjects would be needed to determine whether these differences are influenced by the *APOE* genotype. There are a number of other potential factors that could drive the higher ARIA-E incidence in *APOE* ε4 carriers, for example an increased level of inflammation or differences in microglial function^50–52^.

CAA is present in parenchymal vessels as well as in the leptomeninges^2^. The relative importance of parenchymal and leptomeningeal CAA for the development of ARIA-E is not known. One limitation of this study is that parenchymal CAA has not been studied, and fibrils from parenchymal CAA may behave differently. Work to clarify this could shed further light on ARIA-E mechanisms. The study was also limited by antibody availability, which meant that only lecanemab was the exact therapeutic drug. The other antibodies were generated in-house using publicly available sequences. Finally, the present study focused on antibody binding to CAA Aβ fibrils but it is likely that several other genetic factors, chemical mediators, and cellular functions contribute to the development of ARIA-E^53^.

The efficacy of a therapeutic Aβ antibody depends on its target binding properties^36^, and our data are consistent with the idea that its CAA binding properties could influence its propensity to cause ARIA-E. In addition, development of an in vitro assay of antibody binding to isolated Aβ CAA fibrils could help to predict the likelihood of ARIA-E arising during Aβ immunotherapy. Further characterization of the mechanisms involved will be required in order to minimize risk for ARIA-E and thereby improve the safety of Aβ immunotherapy.

## Materials and methods

### Antibodies

The antibodies employed in this study are described in Table 1. Aducanumab (human IgG1), bapineuzumab (human IgG1), crenezumab (human IgG4), gantenerumab (human IgG1), donanemab (human IgG1), and solanezumab (human IgG1) were transiently expressed and produced from sequences obtained from patents. Lecanemab (human IgG1) was provided by Eisai. All antibodies were stored at – 80 °C.

### Human brain tissue

These studies used human tissue collected by the Netherlands Brain Bank (NBB), Netherlands Institute for Neuroscience, Amsterdam (www.brainbank.nl). The procedures used to collect and analyze the tissue samples were approved and performed in accordance with the ethical standards laid down in the 1964 Declaration of Helsinki. The NBB procedures met all relevant international legal and ethical requirements for scientific research and this research study was approved by the Swedish Ethical Review Authority (no. 2020-00527). The NBB obtained written informed consent for brain autopsy and for the use of tissue and clinical information for research purposes. Post-mortem leptomeningeal tissues were obtained from six subjects with a clinical diagnosis of AD and five NDE subjects (Table 2). Brain temporal cortex samples were also obtained from four AD subjects with high levels of leptomeningeal CAA.

### Immunohistochemical analysis

IHC was performed on fresh frozen leptomeningeal tissues from six AD and five NDE subjects. Tissues were sectioned (8 µm) and mounted onto Superfrost Plus slides (Thermo Fisher). The sections were air-dried in the cryostat for 30 min and stored in a sealed box at − 80 °C until use. The frozen sections were transferred directly to ice-cold 50% acetone for 30 s, followed by 100% acetone for 5 min, and finally 1 × phosphate-buffered saline (PBS) for 5 min before wet-loading into a Ventana robotic platform. The automated staining procedure used a mixture of mouse anti-human Aβ antibodies (6E10 and 4G8; BioLegend) at working concentrations of 1 µg/mL 6E10 and 1 µg/mL 4G8, followed by a horseradish peroxidase (HRP) 3,3ʹ-diaminobenzidine-based detection system (Discovery XT 20 and OmniMap DAB kit, Ventana Medical Systems). The sections were counterstained with hematoxylin before bright-field scanning using a Pannoramic 250 FLASH III slide scanner. The resulting image files were uploaded into Slide Viewer software, version 2.5 (3DHISTECH) and adjusted for optimal brightness and contrast prior to manual assessment of staining.

### Isolation of CAA Aβ fibrils from leptomeningeal tissue

A published protocol^41^ was used to isolate CAA Aβ fibrils from leptomeningeal tissue (Supplementary Fig. 2). Briefly, approximately 50–100 mg tissue was cut into pieces using a scalpel and washed three times with Tris-calcium buffer (20 mM Tris, 138 mM NaCl, 2 mM CaCl_2_, 0.1% (*w/v*) NaN_3_; pH 8.0), followed by centrifugation at 12,000 × *g* for 5 min at 4 °C. The pellet was digested with 5-mg/mL collagenase from *Clostridium histolyticum* (Sigma-Aldrich) overnight at 37 °C in Tris calcium buffer. The digestate was centrifuged at 12,000 × *g* for 5 min at 4 °C and the pellet was washed four times with 500 µL ice-cold wash buffer (50 mM Tris, 10 mM EDTA; pH 8.0), followed by centrifugation at 4 °C for 5 min at 12,000 × *g*. The resultant pellet was resuspended in 500 µL ice-cold dH_2_O and centrifuged for 5 min at 12,000 × *g* at 4 °C. The supernatant, which contained the fibrils, was carefully removed and stored at − 80 °C. This step was repeated another nine times to generate ten water fractions. The final water-insoluble pellet that remained after the tenth extraction was dissolved in 70% formic acid (FA) to disperse fibrils into monomers. As an indicator of the fibril concentration in each of the ten water fractions, Aβ levels were determined using the V-PLEX^®^ Aβ peptide panel 1 (4G8) kit, as described below. For each study subject, fractions with high Aβ levels were pooled to create the CAA Aβ fibril extract that was used in subsequent analyses.

### Isolation of insoluble Aβ fibrils from temporal cortex

Each human temporal cortex sample was placed in tris-buffered saline containing protease and phosphatase inhibitors (Roche) at a 1:2.5 *w/v* ratio. Homogenates were produced using Potter–Elvehjem tissue grinders and stored at − 80 °C. After thawing, the homogenates were further diluted in tris-buffered saline to a final *w/v* ratio of 1:10. They were vortexed (3 × 10 s) before centrifugation at 16,000 × *g* for 60 min at 4 °C. The supernatants (soluble extracts) were removed. The remaining pellets were dissolved in 70% FA before centrifugation at 100,000 × *g* for 60 min at 4 °C. The resultant supernatants were defined as insoluble cortical extracts.

### Characterization of CAA Aβ fibrils

#### CLSM

CAA Aβ fibril samples (20 µL) were loaded onto Nunc™ Glass Bottom Dishes with a 1.2-mm borosilicate glass base insert and allowed to dry for 1 h. Following complete evaporation, 200 µL of Dulbecco’s PBS (Gibco) containing 30-nM pFTAA (kind gift from Peter Nilsson, Linköping University, Sweden) and a 1:500 dilution of Alexa Fluor^®^ 647-conjugated 6E10 (Biolegend) was added onto the glass base insert and incubated for 1 h at room temperature. The glass insert was then washed three times with Dulbecco’s PBS prior to CLSM imaging using a Stellaris 5 system (Leica, Germany) equipped with a white light laser and an HP PLAN APO 20 × /0.75 immersion correction ring objective. pFTAA fluorescence was excited at 488 nm and detected at 490–600 nm; Alexa Fluor^®^ 647-conjugated 6E10 was excited at 647 nm and detected at 651–754 nm. Imaging and acquisition settings were kept consistent throughout and for the negative control, pFTAA and Alexa Fluor® 647-conjugated 6E10 were omitted from the incubation step.

#### Measurement of Aβ38, Aβ40, Aβ42, and AβpE3-40 levels

CAA Aβ fibril samples (6 µL) were added to 14 µL of 100% FA (final concentration: 70% FA) and incubated at room temperature for at least 10 min to disrupt the fibrils. Five microliters of these samples, or of the insoluble temporal cortex extracts, were then neutralized to pH 7.0 ± 0.5 by adding 145 µL of Trizma base/Na_2_HPO_4_ buffer mixed with an equal volume of Diluent 35 (MSD).

The V-PLEX^®^ Aβ peptide panel 1 (4G8) kit (MSD) was used to measure the levels of Aβ38, Aβ40, and Aβ42 using specific pre-coated capture antibodies. Neutralized samples were diluted as necessary in assay diluent prior to adding 25 µL sample and 25 µL anti-Aβ 4G8 SULFO-TAG™ detection antibody to pre-blocked 96-well plates in duplicate. These were incubated for 2 h before washing, adding the MSD read buffer, and analyzing using an MSD sector imager. The levels of Aβ38, Aβ40, and Aβ42 in each sample were calculated from standard curves constructed using peptides provided in the kit. Assay values that were below the lower limit of quantification (LLOQ) were assigned a value of ½ LLOQ.

An enzyme-linked immunosorbent assay kit (IBL) was used to measure AβpE3-40 levels. Neutralized CAA Aβ fibril samples were diluted four-fold in the kit assay buffer before adding them to microtiter plates coated with mouse anti-human Aβ35-40 and incubating overnight. The wells were washed before incubating with HRP-conjugated mouse anti-human AβpE3 (8E1) antibody for 1 h and washed again before adding HRP substrate (3,3ʹ,5,5ʹ-tetramethylbenzidine). The reaction was stopped by adding 0.5-M H_2_SO_4_ before measurement of absorption at 450 nm using a microplate reader. The level of AβpE3-40 in each sample was calculated from a standard curve constructed using the kit peptide standard.

#### IP-MALDI

Magnetic beads (25 μL; Dynabeads M-280 sheep anti-mouse, Thermo Fisher) were incubated with 4 µg of either 6E10 or 4G8 (BioLegend) for 1 h before washing and combining. CAA Aβ fibril extracts were concentrated 20 times before adding 50–117 μL of 100% FA (final concentration: 70% FA) and incubating for 10 min. Each sample was neutralized by the addition of 3.9 mL Trizma base (1 M) immediately prior to adding the magnetic bead-antibody complexes (50 µL) and 20% Triton X-100 in PBS, to achieve a final Triton X-100 concentration of 0.2%. After an overnight incubation at 4 °C, the beads were washed three times; firstly in 0.2% Triton X-100, then in PBS, and finally in 50-mM ammonium bicarbonate. They were then eluted in 100 µL of 70% FA. The resultant eluate was dried in a vacuum centrifuge and stored at −80 °C. Prior to MALDI analysis, the samples were reconstituted in 5 μL dH_2_O containing 20% acetonitrile and 0.1% FA and prepared using the seed layer method with α-cyano-4-hydroxycinnamic acid as the matrix, as described previously^9^. Analysis was performed on a Bruker Daltonics rapifleX MALDI-TOF instrument by acquiring an average of 10,000 shots for each spectrum (2,000 at a time using random walk mode). Data were analyzed using an in-house script to match the masses of the observed peaks to those of known Aβ peptides. For each spectrum, each peak area was normalized to the sum of all Aβ peak areas in the same spectrum before further analysis. This produced a profile of the Aβ peptides present in each sample, and indicated their relative abundance.

### In vitro measurement of antibody binding to CAA Aβ fibrils

#### IP

Automated IP was performed using a KingFisher Apex instrument (Thermo Fisher). CAA Aβ fibrils isolated from four AD subjects were diluted in 1% Blocker A (MSD) in PBS by 1:20 (except for AD021, which was diluted 1:1000 because it contained a high concentration of Aβ) before adding 15 µL to 1% Blocker A (135 µL) that had been supplemented with the test antibody to produce final concentrations of 0.7, 7, 70, 700, 3500 or 17,500 pM. After incubating at 22 °C for 2 h, 450 µg magnetic protein A beads (Thermo Fisher) were added and incubated with the antibody/Aβ complexes for 30 min at 22 °C. The beads were then washed twice in 1% Blocker A before elution in 70% FA. The eluates were diluted and neutralized to pH 7.0 ± 0.5 in Trizma base/Na_2_HPO_4_ buffer mixed with an equal volume of Diluent 35 (MSD), representing a total dilution of 1:30, before analysis of Aβ38, Aβ40, and Aβ42 using the V-PLEX^®^ Aβ peptide panel 1 (4G8) kit, as described above. EC_50_ values were calculated by plotting the bound Aβ40 levels against the IP antibody concentration and using GraphPad Prism to analyze nonlinear regression with sigmoidal dose response (variable slope).

#### SPR

SPR was performed using a Biacore™ 8 K + instrument (Cytiva). The CAA Aβ fibrils (3–6 µL diluted in 10 mM acetate buffer; pH 4.5) were immobilized on a CM5 chip using the “immobilize low levels” surface preparation method specified in the Biacore™ 8 K Control Software and standard amine coupling. Single cycle kinetic analyses were then performed using five concentrations of each antibody, injected over the immobilized CAA Aβ fibrils. The injection time for each concentration was 2 min and the dissociation time was 20 min. The surface was regenerated between each cycle using a 30-s injection of 3-M MgCl_2_. A blank cycle, with running buffer, was performed in between each three-fold antibody dilution series (6.2, 18.5, 55.6, 167, and 500 nM). The data were fitted to a bivalent analyte model. The affinities are reported as K_*D1*_ and should be read as apparent K_*D*_.

#### Direct MSD measurement

CAA Aβ fibrils were diluted in dH_2_O (1:200 for AD021 and 1:25 for the other subjects, to achieve a similar amount of CAA fibrils) before adding 12 µL to 384-well standard MSD plates and incubating overnight at 4 °C. The plates were washed with PBS containing Tween 20 (0.1%) before blocking with 1% Blocker A (MSD) for 60–180 min, and washed again prior to incubation with the test antibodies (10,000 ng/mL) for 60 min. The plates were washed and incubated with an anti-human SULFO-TAG™ detection antibody (1:1000 dilution; MSD) for 60 min before adding the MSD read buffer and analyzing the signal using an MSD sector imager. All incubations were performed on a shaking plate (900 rpm) at RT. The non-specific signal generated by test antibody binding to uncoated wells was subtracted from each raw signal reading.

### Statistics

For the CAA Aβ fibril binding IP experiments, the median EC_50_ value of three IP analyses per subject and antibody was log-transformed and used in subsequent statistical analyses. For direct MSD measurements, the individual rank scores were used in subsequent statistical analyses. IP, MSD, and SPR data were analyzed by one-way repeated ANOVA, with the relevant antibody as within-subject factor. The alpha level was set at 0.05 and a significant ANOVA test was followed by Tukey’s unequal N honest significant difference post-hoc testing (IP and SPR data) or Tukey’s post-hoc test (MSD data).

### Supplementary Information

Supplementary Information.

## Data Availability

The datasets generated and/or analyzed during the current study are available from the corresponding author on request.

## References

[1] Murphy MP, LeVine H (2010). Alzheimer's disease and the amyloid-beta peptide. J. Alzheimer’s Dis..

[2] Greenberg SM (2020). Cerebral amyloid angiopathy and Alzheimer disease—One peptide, two pathways. Nat. Rev. Neurol..

[3] Charidimou A (2022). The Boston criteria version 2.0 for cerebral amyloid angiopathy: A multicentre, retrospective, MRI-neuropathology diagnostic accuracy study. Lancet Neurol..

[4] Charidimou A (2017). Emerging concepts in sporadic cerebral amyloid angiopathy. Brain.

[5] Koemans EA (2023). Progression of cerebral amyloid angiopathy: A pathophysiological framework. Lancet Neurol..

[6] Thal DR (2002). Two types of sporadic cerebral amyloid angiopathy. J. Neuropathol. Exp. Neurol..

[7] Attems J, Lintner F, Jellinger KA (2004). Amyloid beta peptide 1–42 highly correlates with capillary cerebral amyloid angiopathy and Alzheimer disease pathology. Acta Neuropathol..

[8] Oshima K (2006). Relative paucity of tau accumulation in the small areas with abundant Abeta42-positive capillary amyloid angiopathy within a given cortical region in the brain of patients with Alzheimer pathology. Acta Neuropathol..

[9] Gkanatsiou E (2019). A distinct brain beta amyloid signature in cerebral amyloid angiopathy compared to Alzheimer's disease. Neurosci. Lett..

[10] Rajpoot J (2022). Insights into cerebral amyloid angiopathy Type 1 and Type 2 from comparisons of the fibrillar assembly and stability of the Aβ40-Iowa and Aβ40-Dutch peptides. Biochemistry.

[11] Drzezga A (2009). Effect of APOE genotype on amyloid plaque load and gray matter volume in Alzheimer disease. Neurology.

[12] Grimmer T (2010). Progression of cerebral amyloid load is associated with the apolipoprotein E ε4 genotype in Alzheimer's disease. Biol. Psychiatry.

[13] Nie H (2019). Apolipoprotein E gene polymorphisms are risk factors for spontaneous intracerebral hemorrhage: A systematic review and meta-analysis. Curr. Med. Sci..

[14] Budd Haeberlein, S. *et al*. Two randomized phase 3 studies of aducanumab in early Alzheimer's disease. *J. Prev. Alzheimers Dis*. **9**, 197–210. 10.14283/jpad.2022.30 (2022). 10.14283/jpad.2022.30 35542991

[15] Salloway S (2014). Two phase 3 trials of bapineuzumab in mild-to-moderate Alzheimer's disease. N. Engl. J. Med..

[16] Sims JR (2023). Donanemab in early symptomatic Alzheimer disease: The TRAILBLAZER-ALZ 2 randomized clinical trial. JAMA.

[17] van Dyck CH (2023). Lecanemab in early Alzheimer's disease. N. Engl. J. Med..

[18] Klein G (2019). Gantenerumab reduces amyloid-β plaques in patients with prodromal to moderate Alzheimer's disease: A PET substudy interim analysis. Alzheimers Res. Ther..

[19] Ostrowitzki S (2012). Mechanism of amyloid removal in patients with Alzheimer disease treated with gantenerumab. Arch. Neurol..

[20] Boche D (2008). Consequence of Abeta immunization on the vasculature of human Alzheimer's disease brain. Brain.

[21] Sperling RA (2011). Amyloid-related imaging abnormalities in amyloid-modifying therapeutic trials: Recommendations from the Alzheimer's Association Research Roundtable Workgroup. Alzheimers Dement..

[22] Carare RO (2020). Clearance of interstitial fluid (ISF) and CSF (CLIC) group-part of Vascular Professional Interest Area (PIA): Cerebrovascular disease and the failure of elimination of Amyloid-β from the brain and retina with age and Alzheimer's disease—Opportunities for therapy. Alzheimers Dement..

[23] Hampel H (2023). Amyloid-related imaging abnormalities (ARIA): Radiological, biological and clinical characteristics. Brain..

[24] Lemere, C. What we have learned about ARIA in anti-amyloid antibody treatment in mice and the implications for AD clinical trials. In *16th Clinical Trials on Alzhimer's Disease (CTAD), Boston* (2023).

[25] Salloway S (2022). Amyloid-related imaging abnormalities in 2 Phase 3 studies evaluating aducanumab in patients with early Alzheimer disease. JAMA Neurol..

[26] Joseph-Mathurin N (2022). Amyloid-related imaging abnormalities in the DIAN-TU-001 trial of gantenerumab and solanezumab: Lessons from a trial in dominantly inherited Alzheimer disease. Ann. Neurol..

[27] Salloway S (2021). A trial of gantenerumab or solanezumab in dominantly inherited Alzheimer's disease. Nat. Med..

[28] Bateman RJ (2023). Two phase 3 trials of gantenerumab in early Alzheimer's disease. N. Engl. J. Med..

[29] Cummings JL (2018). ABBY: A phase 2 randomized trial of crenezumab in mild to moderate Alzheimer disease. Neurology.

[30] Racke MM (2005). Exacerbation of cerebral amyloid angiopathy-associated microhemorrhage in amyloid precursor protein transgenic mice by immunotherapy is dependent on antibody recognition of deposited forms of amyloid beta. J. Neurosci..

[31] Doody RS (2014). Phase 3 trials of solanezumab for mild-to-moderate Alzheimer's disease. N. Engl. J. Med..

[32] Honig LS (2018). Trial of solanezumab for mild dementia due to Alzheimer's disease. N. Engl. J. Med..

[33] Bohrmann B (2012). Gantenerumab: A novel human anti-Aβ antibody demonstrates sustained cerebral amyloid-β binding and elicits cell-mediated removal of human amyloid-β. J. Alzheimers Dis..

[34] Sevigny J (2016). The antibody aducanumab reduces Aβ plaques in Alzheimer's disease. Nature.

[35] Mintun MA (2021). Donanemab in early Alzheimer's disease. N. Engl. J. Med..

[36] Söderberg L (2023). Lecanemab, aducanumab, and gantenerumab—binding profiles to different forms of amyloid-beta might explain efficacy and side effects in clinical trials for Alzheimer's disease. Neurotherapeutics.

[37] Rinne JO (2010). 11C-PiB PET assessment of change in fibrillar amyloid-beta load in patients with Alzheimer's disease treated with bapineuzumab: A phase 2, double-blind, placebo-controlled, ascending-dose study. Lancet Neurol..

[38] Meilandt WJ (2019). Characterization of the selective in vitro and in vivo binding properties of crenezumab to oligomeric Aβ. Alzheimers Res. Ther..

[39] Farlow M (2012). Safety and biomarker effects of solanezumab in patients with Alzheimer's disease. Alzheimers Dement..

[40] Klingstedt T (2013). The structural basis for optimal performance of oligothiophene-based fluorescent amyloid ligands: Conformational flexibility is essential for spectral assignment of a diversity of protein aggregates. Chemistry.

[41] Kollmer M (2019). Cryo-EM structure and polymorphism of Aβ amyloid fibrils purified from Alzheimer's brain tissue. Nat. Commun..

[42] Miller DL (1993). Peptide compositions of the cerebrovascular and senile plaque core amyloid deposits of Alzheimer's disease. Arch. Biochem. Biophys..

[43] Gerth J (2018). Modified amyloid variants in pathological subgroups of *β* -amyloidosis. Ann. Clin. Transl. Neurol..

[44] Swanson CJ (2021). A randomized, double-blind, phase 2b proof-of-concept clinical trial in early Alzheimer's disease with lecanemab, an anti-Aβ protofibril antibody. Alzheimers Res. Ther..

[45] Lannfelt, L. *et al*. Binding profiles of lecanemab and donanemab to different amyloid-beta species. *JPAD* **S26** (2023).

[46] Piazza F (2013). Anti-amyloid β autoantibodies in cerebral amyloid angiopathy-related inflammation: Implications for amyloid-modifying therapies. Ann. Neurol..

[47] Piazza F (2022). Association of microglial activation with spontaneous ARIA-E and CSF levels of anti-Aβ autoantibodies. Neurology.

[48] Antolini L (2021). Spontaneous ARIA-like events in cerebral amyloid angiopathy-related inflammation: A multicenter prospective longitudinal cohort study. Neurology.

[49] Crehan H (2020). Effector function of anti-pyroglutamate-3 Aβ antibodies affects cognitive benefit, glial activation and amyloid clearance in Alzheimer's-like mice. Alzheimers Res. Ther..

[50] Lin YT (2018). APOE4 causes widespread molecular and cellular alterations associated with Alzheimer's disease phenotypes in human iPSC-derived brain cell types. Neuron.

[51] Liu CC (2023). Cell-autonomous effects of APOE4 in restricting microglial response in brain homeostasis and Alzheimer's disease. Nat. Immunol..

[52] Yin Z (2023). APOE4 impairs the microglial response in Alzheimer's disease by inducing TGFβ-mediated checkpoints. Nat. Immunol..

[53] Lowe SL (2021). Donanemab (LY3002813) dose-escalation study in Alzheimer's disease. Alzheimers Dement..

[54] Ostrowitzki S (2022). Evaluating the safety and efficacy of crenezumab vs placebo in adults with early Alzheimer disease: Two phase 3 randomized placebo-controlled trials. JAMA Neurol..

[55] Thal DR, Rüb U, Orantes M, Braak H (2002). Phases of A beta-deposition in the human brain and its relevance for the development of AD. Neurology.

