# Supplementary material for: Amyloid-beta antibody binding to cerebral amyloid angiopathy fibrils and risk for amyloid-related imaging abnormalities
Source: Sci Rep. 2024 May 13;14:10868. doi: 10.1038/s41598-024-61691-2 (PMC11091209; doi:10.1038/s41598-024-61691-2)
Supplement: Supplementary file 1 — Supplementary Information. [file 41598_2024_61691_MOESM1_ESM.pdf]

Supplementary information

Amyloid-beta antibody binding to cerebral amyloid angiopathy fibrils and risk for amyloid-related imaging abnormalities

Linda Söderberg[1], Malin Johannesson[1], Eleni Gkanatsiou[1], Patrik Nygren[1], Nicolas Fritz[1], Olof Zachrisson[1], Adeline Rachalski[1], Anne-Sophie Svensson[1], Emily Button[1], Giacomo Dentoni[1], Gunilla Osswald[1], Lars Lannfelt[1, 2], Christer Möller[1]

[1]BioArctic AB, Warfvinges väg 35, 112 51, Stockholm, Sweden.

[2]Dept. of Public Health/Geriatrics, Uppsala University, 751 85, Uppsala, Sweden.

Supplementary Fig. 1

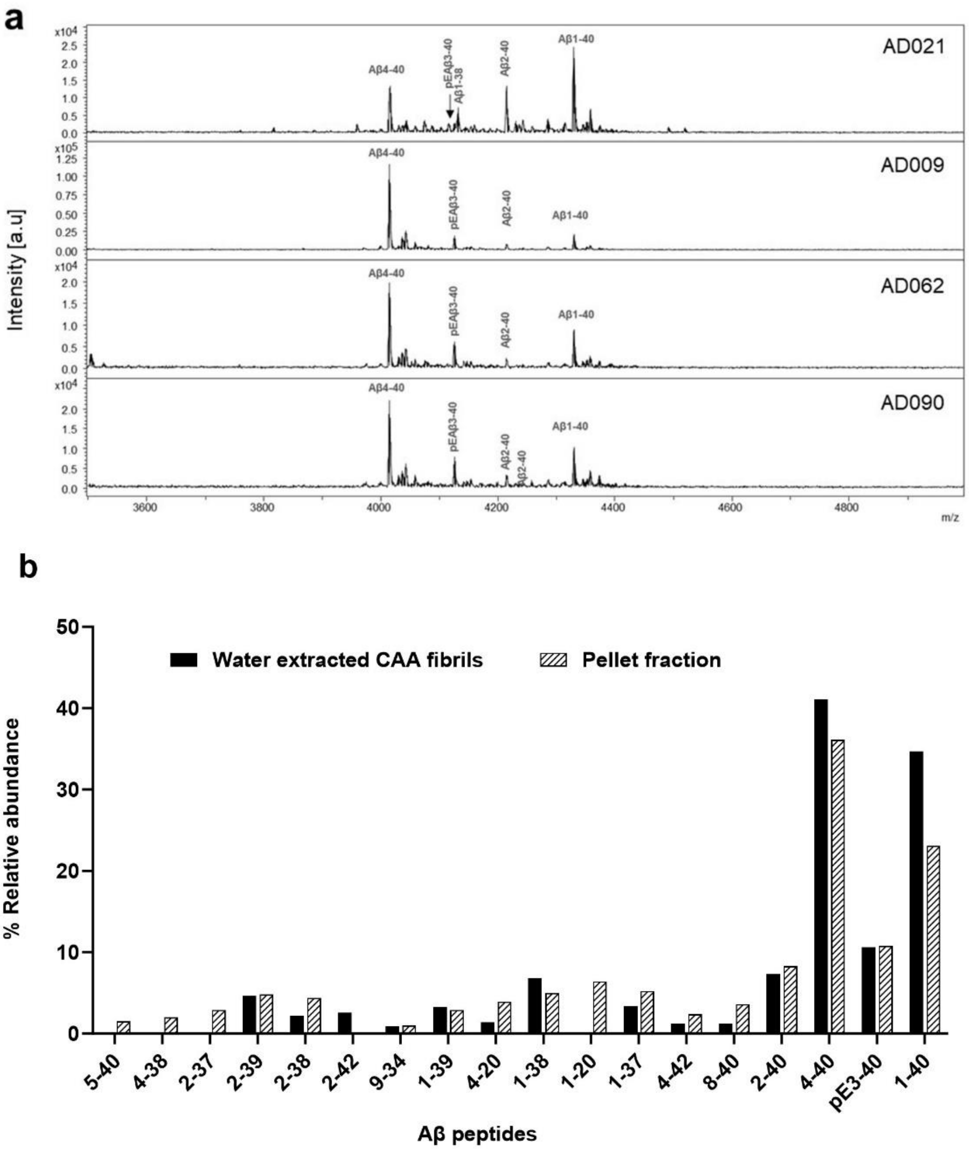

Supplementary Figure 1. IP-MALDI analyses of the FA-extracted pellet fractions from leptomeningeal tissues of AD subjects. **a,** Mass spectra for the indicated study subjects showing Aβ-assigned peaks. **b,** The relative abundance of the Aβ peptides identified in each pellet fraction sample (average of all subjects in **a**), as compared with the water-soluble extract from the same individual (data presented in Fig. 4).

Supplementary Fig. 2

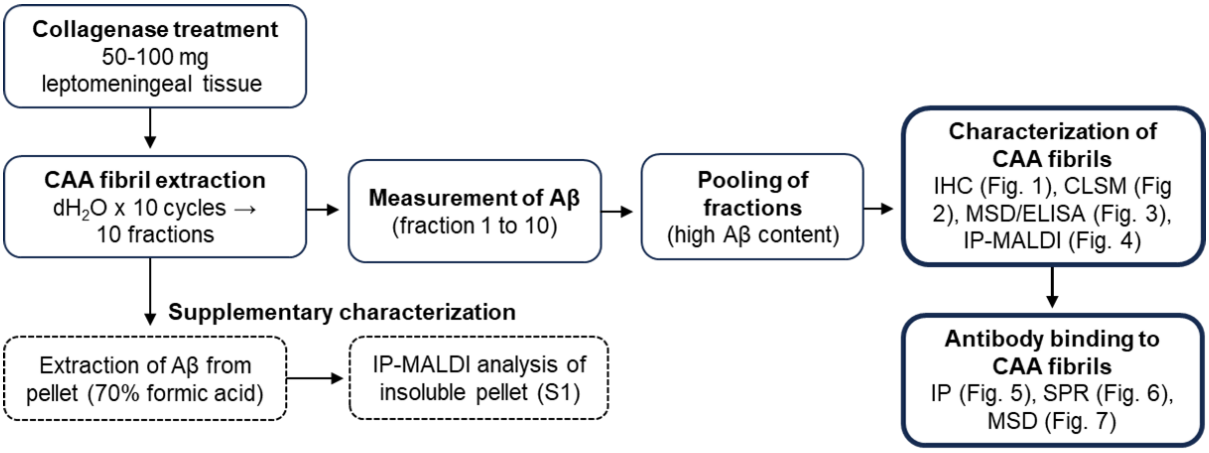

Supplementary Figure 2. Schematic outline of the steps used to isolate and characterize CAA Aβ fibrils from human leptomeningeal tissue. Repeated water extraction of the tissue generated ten water fractions. The Aβ content of each fraction was analyzed using the V-PLEX® Aβ peptide panel 1 (4G8) assay (MSD). Fractions with high Aβ levels (usually fractions 2 to 6) were pooled, generating a representative sample from each subject for use in subsequent analyses. In a supplementary characterization, the final pellet that remained after all the water extractions had been completed was extracted in 70% formic acid prior to peptide characterization using immunoprecipitation (IP) matrix-assisted laser desorption/ionization time-of-flight mass spectrometry (MALDI). IHC, immunohistochemistry; ELISA, enzyme-linked immunosorbent assay; CLSM, confocal laser scanning microscopy; SPR, surface plasmon resonance.

Supplementary Table 1: SPR kinetics of antibody binding to CAA Aβ fibrils

AD009			
**Antibody**	**k_{a1} ($M^{-1}s^{-1}$)**	**k_{d1} (s^{-1})**	**K_{D1} (nM)**
Aducanumab	$3.36 \pm 1.20 \times 10^5$	$1.14 \pm 0.28 \times 10^{-1}$	358 ± 71.7
Bapineuzumab	$6.72 \pm 7.86 \times 10^5$	$1.31 \pm 0.83 \times 10^{-3}$	5.96 ± 4.96
Crenezumab	ND	ND	ND
Donanemab	$1.25 \pm 0.37 \times 10^4$	$3.08 \pm 0.39 \times 10^{-2}$	728 ± 119
Gantenerumab	$6.46 \pm 1.41 \times 10^4$	$3.01 \pm 0.71 \times 10^{-2}$	471 ± 57.0
Lecanemab	$5.36 \pm 2.73 \times 10^4$	$3.91 \pm 1.30 \times 10^{-2}$	764 ± 134
Solanezumab	ND	ND	ND

AD062			
**Antibody**	**k_{a1} ($M^{-1}s^{-1}$)**	**k_{d1} (s^{-1})**	**K_{D1} (nM)**
Aducanumab	$8.53 \pm 3.47 \times 10^4$	$1.03 \pm 0.41 \times 10^{-1}$	1220 ± 282
Bapineuzumab	ND	ND	ND
Crenezumab	ND	ND	ND
Donanemab	ND	ND	ND
Gantenerumab	$3.29 \pm 0.40 \times 10^4$	$4.59 \pm 0.48 \times 10^{-2}$	1400 ± 92.6
Lecanemab	ND	ND	ND
Solanezumab	ND	ND	ND

AD090			
**Antibody**	**k_{a1} ($M^{-1}s^{-1}$)**	**k_{d1} (s^{-1})**	**K_{D1} (nM)**
Aducanumab	$1.33 \pm 1.00 \times 10^6$	$5.48 \pm 3.01 \times 10^{-2}$	54.9 ± 31.3
Bapineuzumab	$9.93 \pm 10.1 \times 10^5$	$9.10 \pm 4.73 \times 10^{-5}$	0.17 ± 0.13
Crenezumab	ND	ND	ND
Donanemab	$2.28 \pm 1.23 \times 10^4$	$8.29 \pm 2.84 \times 10^{-3}$	465 ± 367
Gantenerumab	$7.71 \pm 4.23 \times 10^4$	$2.29 \pm 0.89 \times 10^{-2}$	347 ± 132
Lecanemab	$7.96 \pm 1.44 \times 10^4$	$8.35 \pm 1.14 \times 10^{-2}$	1060 ± 160
Solanezumab	ND	ND	ND

AD021			
**Antibody**	**k_{a1} ($M^{-1}s^{-1}$)**	**k_{d1} (s^{-1})**	**K_{D1} (nM)**
Aducanumab	$2.52 \pm 0.18 \times 10^5$	$1.38 \pm 0.08 \times 10^{-1}$	548 ± 36.7
Bapineuzumab	$3.33 \pm 0.21 \times 10^5$	$9.83 \pm 4.70 \times 10^{-5}$	0.30 ± 0.16
Crenezumab	$8.30 \pm 2.62 \times 10^3$	$1.53 \pm 1.02 \times 10^{-2}$	2020 ± 1540
Donanemab	$2.53 \pm 1.43 \times 10^4$	$3.02 \pm 1.18 \times 10^{-2}$	1260 ± 192
Gantenerumab	$7.49 \pm 0.58 \times 10^4$	$2.92 \pm 0.34 \times 10^{-2}$	390 ± 22.0
Lecanemab	$1.28 \pm 0.11 \times 10^5$	$2.18 \pm 0.12 \times 10^{-2}$	170 ± 9.85
Solanezumab	ND	ND	ND

Data shown as mean ± SD. ND: not detected.
k_{a1}: association rate constant; k_{d1}: dissociation rate constant; K_{D1}: dissociation constant.
